# Targeting cardiac fibrosis with chimeric antigen receptor macrophages

**DOI:** 10.1038/s41421-024-00718-4

**Published:** 2024-08-13

**Authors:** Zibei Gao, Lei Yan, Jufeng Meng, Zhengkai Lu, Kaixin Ge, Zhen Jiang, Teng Feng, Haopeng Wang, Chen Liu, Juan Tang, Hui Zhang

**Affiliations:** 1 School of Life Science and Technology & Shanghai Clinical Research and Trial Center, ShanghaiTech University, Shanghai, China; 2 State Key Laboratory of Cardiovascular Disease and Medical Innovation Center, Shanghai East Hospital, Frontier Science Center for Stem Cell Research, School of Life Science and Technology, Tongji University, Shanghai, China; 3 Department of Cardiac Surgery, Zhongshan Hospital, Fudan University, Shanghai, China; 4 State Key Laboratory of Advanced Medical Materials and Devices, ShanghaiTech University, Shanghai, China

**Keywords:** Mechanisms of disease, Heart stem cells

Dear Editor,

Cardiac fibrosis, a significant global health issue associated with nearly all forms of heart disease, is characterized by excessive extracellular matrix (ECM) deposition within the myocardium, leading to reduced tissue compliance and accelerating the progression to heart failure^1^. In response to myocardial injury, resident cardiac fibroblasts are recruited to injured zones, where they become highly activated fibroblasts (myofibroblasts), enhancing the secretion of ECM proteins^2^. Genetic ablation of myofibroblasts following injury reduces ECM production, alleviates cardiac fibrosis, and improves heart function^3,4^. However, clinical interventions and therapies targeting cardiac myofibroblasts remain limited.

Recent progress in immunotherapeutic ablation of myofibroblasts has yielded promising results. Adoptive transfer of T cells expressing a chimeric antigen receptor (CAR) against fibroblast activation protein (FAP), a cell-surface glycoprotein strongly expressed by myofibroblasts in damaged hearts^5–7^, significantly reduced cardiac fibrosis and improved heart function in a mouse model of hypertensive cardiac injury induced by angiotensin II and phenylephrine (AngII/PE)^7^. Moreover, in vivo reprogramming of CAR T cells via delivery of modified mRNA encoding a CAR targeting FAP in T cell-targeted lipid nanoparticles, also mitigated cardiac fibrosis and improved heart function following AngII/PE-induced heart injury^8^. These results suggest that CAR T cells targeting myofibroblasts have therapeutic potential for cardiac fibrosis treatment.

CAR T cell therapy encounters numerous challenges in tumor treatment, such as limited infiltration into dense extracellular matrix, exhaustion in the tumor microenvironment, off-target effects, and heterogeneity within the tumor^9^. Recently, CAR-expressing macrophages (CAR-M) have garnered increasing attention in the oncology field. Compared to CAR T cells, CAR-M cells have superior infiltration capabilities, allowing them to penetrate dense tissues more effectively. Beyond phagocytosis, macrophages can also directly release cytotoxic granules and cytokines, possess antigen-presenting ability, and regulate other immune cells. Furthermore, their limited circulation time may pose lower risks and improve safety during the treatment process. However, the application of CAR-M cells in cardiovascular diseases remains unexplored. The potential for CAR-M cells to eliminate myofibroblasts and mitigate cardiac fibrosis in injured hearts remains uncertain.

**Fig. 1:**
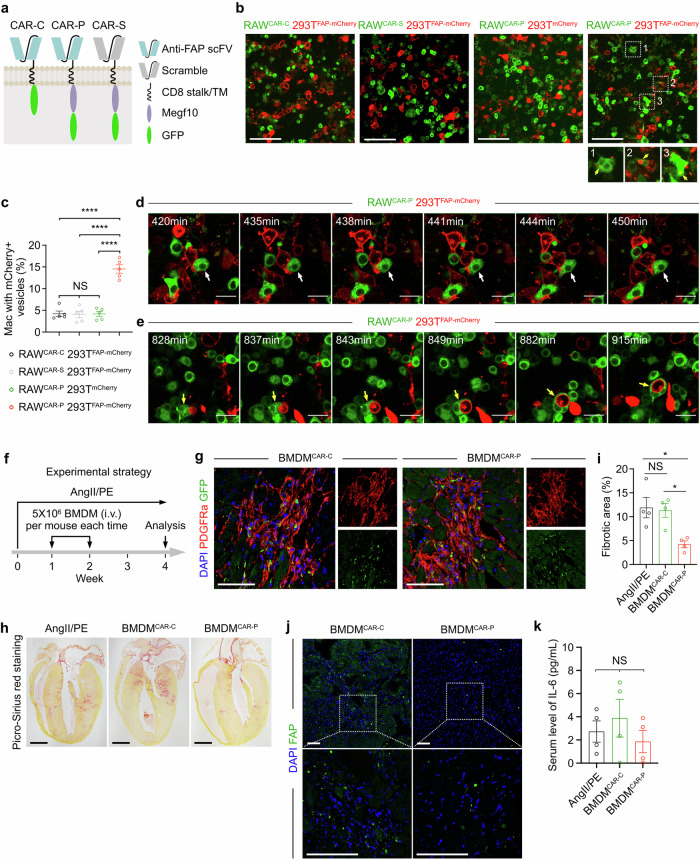
FAP CAR-M cells reduce cardiac fibrosis. **a** Design strategy of the CAR-C, CAR-S, and CAR-P. **b** Fully internalized mCherry^+^ vesicles (yellow arrows) identified in the RAW^CAR-P^ cells co-cultured with 293T^FAP-mCherry^ target cells. Scale bars, 100 µm. **c** Percentage of macrophages with internalized mCherry^+^ vesicles. Each group contained five independent experiments. NS, non-significant; ****P < 0.0001. **d** Live-cell imaging of a RAW^CAR-P^ cell (white arrow) nibbling a 293T^FAP-mCherry^ target cell. Scale bars, 25 µm. **e** Live-cell imaging of a RAW^CAR-P^ cell (yellow arrow) engulfing a whole 293T^FAP-mCherry^ cell. Scale bars, 25 µm. **f** Experimental timeline of the CAR-M treatment strategy. Mice were continuously administered AngII/PE to induce cardiac fibrosis. BMDM^CAR^ cells were injected intravenously one and two weeks after the initial AngII/PE treatment. The hearts were harvested for analysis four weeks after the initial AngII/PE treatment. **g** Immunostaining for GFP and PDGFRa demonstrating BMDM^CAR^ accumulation in hearts four weeks following the initial AngII/PE treatment. Scale bars, 200 µm. **h** Picro-Sirius staining to evaluate the level of cardiac fibrosis four weeks after the initial AngII/PE treatment. Scale bars, 2 mm. **i** Percentage of fibrotic area in the hearts. Each group contained four samples. NS, non-significant; *P < 0.05. **j** FAP staining of heart sections from mice four weeks after the initial AngII/PE treatment. Scale bars, 100 µm. **k** Enzyme-linked immunosorbent assay illustrating the serum level of IL-6 in the mice four weeks after the initial AngII/PE treatment. Each group contained four samples. Differences in **c**, **i**, and **k** were analyzed using one-way ANOVA. Each image represents four or five independent experiments.

FAP is reported as a marker of cardiac myofibroblasts after injury, with undetectable expression in other cardiac cell types (Supplementary Fig. S1)^7^. Few FAP^+^ cells were observed in other organs of mice treated with AngII/PE (Supplementary Fig. S2). Therefore, we targeted FAP^+^ fibroblasts and produced a CAR strategy for phagocytosis (CAR-P), aiming to direct macrophages to engulf FAP^+^ myofibroblasts (Fig. 1a; Supplementary Table S1). The CAR-P molecule includes an extracellular single-chain antibody variable fragment (scFV) recognizing FAP^10^, a CD8 transmembrane domain, and a cytoplasmic domain (Megf10 intracellular domain)^11,12^ facilitating phagocytosis, followed by a GFP tag (Fig. 1a, right). Additionally, we developed a control CAR molecule (CAR-C) lacking the phagocytic signaling domain and a scrambled CAR molecule (CAR-S) with a scrambled scFV, both serving as comparisons to CAR-P (Fig. 1a).

To investigate the feasibility of CAR-P, we produced macrophages expressing CAR-P and examined their capacity to engulf FAP^+^ target cells in vitro. CAR-P, CAR-C, and CAR-S molecules were stably transduced into RAW264.7 (RAW) murine macrophages using lentiviral constructs (Supplementary Fig. S3a–d). Similarly, FAP-P2A-mCherry-CaaX and mCherry-CaaX were introduced into HEK293T (293 T) cells (Supplementary Fig. S3e). The fusion of FAP with mCherry via P2A enabled mCherry fluorescence to be a proxy for FAP expression. The CaaX motif ensured the localization of mCherry to the cellular membrane. Co-culture experiments with CAR-expression RAW macrophages and beads revealed that CAR expression did not significantly impact the general phagocytic role of RAW macrophages (Supplementary Fig. S4).

Next, we co-cultured CAR-expressing RAW macrophages with 293 T target cells to evaluate macrophage phagocytosis. We found that a significant proportion of RAW^CAR-P^ macrophages contained one or more fully internalized mCherry^+^ vesicles following co-culture with 293T^FAP-mCherry^ cells (Fig. 1b). In comparison, fewer mCherry^+^ vesicles were detected in RAW^CAR-P^ macrophages co-cultured with 293T^mCherry^ cells, and in RAW^CAR-C^ or RAW^CAR-S^ macrophages co-cultured with 293T^FAP-mCherry^ cells (Fig. 1b, c). To confirm that the internalized vesicles originated from target cells engulfed by macrophages, we conducted live-cell imaging and directly observed the nibbling process of RAW^CAR-P^ macrophages towards 293T^FAP-mCherry^ cells (Fig. 1d; Supplementary Video S1). In some instances, RAW^CAR-P^ macrophages engulfed entire 293T^FAP-mCherry^ cells (Fig. 1e; Supplementary Video S2). We observed that CAR-P enhanced the capacity of RAW macrophages to engulf FAP-overexpressing mouse embryonic fibroblasts (Supplementary Fig. S5) and the ability of THP-1 monocyte-derived human macrophages to engulf 293T^FAP-mCherry^ cells (Supplementary Fig. S6). Overall, these data demonstrate the CAR-P-mediated phagocytosis of FAP^+^ target cells by macrophages in vitro.

We next aimed to utilize CAR-M to reduce cardiac fibrosis following heart exposure to AngII/PE. To achieve this, we generated CAR-M by overexpressing CAR-C or CAR-P in bone marrow-derived macrophages (BMDM) via lentiviral transduction in vitro, while maintaining macrophage marker CD68 expression (Supplementary Fig. S7). BMDM^CAR-C^ and BMDM^CAR-P^ were then introduced into mice via adoptive transfer one and two weeks following AngII/PE initiation (Fig. 1f). Both BMDM^CAR-C^ and BMDM^CAR-P^ cells were detectable in the hearts three days after the final BMDM injection (Supplementary Fig. S8), and they infiltrated fibrotic areas of the myocardium four weeks after the initial AngII/PE treatment (Fig. 1g). Notably, few BMDM^CAR-C^ and BMDM^CAR-P^ cells were found in non-cardiac organs, including the livers, lungs, kidneys, intestines, and testes (Supplementary Fig. S9). Upon examination of cardiac fibrosis four weeks after the initial AngII/PE treatment, we identified that BMDM^CAR-P^ treatment reduced cardiac fibrosis compared to the control groups (Fig. 1h, i). Notably, there was a reduction in the number of FAP^+^ cells detected in BMDM^CAR-P^-treated hearts (Fig. 1j).

We examined potential toxicities associated with FAP CAR-M treatment. Histological analysis of non-cardiac organs uncovered no significant differences in mice receiving FAP CAR-M cell therapy (Supplementary Fig. S10). Compared to the AngII/PE-only group, BMDM^CAR-P^ treatment did not significantly elevate serum cytokine levels, but displayed a lower concentration of the inflammatory chemokine C-C motif chemokine ligand 5 (Supplementary Fig. S11). Moreover, we did not identify significant differences in the serum level of IL-6 in the BMDM^CAR-P^ treated mice (Fig. 1k). Immunostaining for CD3, CD9, and myeloperoxidase indicated that BMDM^CAR-P^ treatment did not enhance additional immune cell infiltration into the heart four weeks following the initial AngII/PE treatment (Supplementary Fig. S12).

Most BMDM^CAR-P^ cells exhibit a Ly6C^low^ and ARG1^+^ phenotype (Supplementary Fig. S13a–c), indicating their M2-like characteristics in vitro. Following adoptive transfer, the majority of BMDM^CAR-P^ cells retained their ARG1 expression four weeks following the initial AngII/PE treatment (Supplementary Fig. S13d, e), suggesting minimal transformation into M1 macrophages in vivo.

We also evaluated the toxicity of CAR-M treatment over a prolonged duration (Supplementary Fig. S14a). Following nine weeks of AngII/PE treatment, BMDM^CAR-P^ therapy did not significantly influence the body weight, survival rate, or histological morphology of non-cardiac organs (Supplementary Fig. S14b–d). However, mice administered BMDM^CAR-P^ exhibited improved heart function, including an increase in ejection fraction, fractional shortening, and a reduction in end-systolic volume and left ventricular end-systolic dimension (Supplementary Fig. S14e). Few BMDM^CAR-C^ or BMDM^CAR-P^ cells were identified in the hearts or blood nine weeks post the initial AngII/PE treatment (Supplementary Fig. S15). Additionally, no significant differences in serum cytokine levels, aside from IL-13, were observed in BMDM^CAR-P^-treated mice (Supplementary Fig. S16). Similarly, no additional immune cell infiltration was observed in the heart nine weeks after the initial AngII/PE treatment (Supplementary Fig. S17).

In conclusion, our study demonstrates that the adoptive transfer of BMDM^CAR-P^ cells targeting FAP^+^ myofibroblasts reduces cardiac fibrosis and improves heart function in the AngII/PE-induced heart injury model. These findings establish a proof-of-concept for the clinical application of CAR-M cells targeting cardiac fibrosis, pioneering a novel research direction in cardiovascular medicine and establishing the foundation for further investigation and advancement. Some limitations still exist, such as the lack of in vivo evidence of BMDM^CAR-P^ cells directly targeting FAP^+^ cells. However, given current technological limitations, it is challenging to directly monitor the engulfment process of CAR-M towards FAP^+^ cells in mouse hearts. Additional investigation should be conducted to optimize the CAR-M strategy. For instance, besides Megf10, several other phagocytic receptors have been reported^9,11,12^, which could potentially function as more efficient mediators for myofibroblast clearance. Furthermore, optimizing the antigen target of CAR-M may yield more substantial reductions in fibrotic remodeling, fostering improved heart function post-injury.

### Supplementary information

Supplementary Information

Supplementary Video S1

Supplementary Video S2
