# Supplementary material for: Targeting cardiac fibrosis with chimeric antigen receptor macrophages
Source: Cell Discov. 2024 Aug 13;10:86. doi: 10.1038/s41421-024-00718-4 (PMC11319452; doi:10.1038/s41421-024-00718-4)
Supplement: Supplementary file 1 — Supplementary Information [file 41421_2024_718_MOESM1_ESM.pdf]

Supplementary Information for:

Targeting cardiac fibrosis with chimeric antigen receptor macrophages

Zibei Gao[1], Lei Yan[1], Jufeng Meng[1], Zhengkai Lu[2], Kaixin Ge[3], Zhen Jiang[1], Teng Feng[1], Haopeng Wang[1,4], Chen Liu[3], Juan Tang[2], and Hui Zhang[1,4*]

[1]School of Life Science and Technology & Shanghai Clinical Research and Trial Center, ShanghaiTech University, Shanghai, China

[2]State Key Laboratory of Cardiovascular Disease and Medical Innovation Center, Shanghai East Hospital, Frontier Science Center for Stem Cell Research, School of Life Science and Technology, Tongji University, Shanghai, China.

[3]Department of Cardiac Surgery, Zhongshan Hospital, Fudan University, Shanghai, China.

[4]State Key Laboratory of Advanced Medical Materials and Devices, ShanghaiTech University, Shanghai, China

These authors contributed equally: Zibei Gao, Lei Yan, Jufeng Meng.

[*]Correspondence: Chen Liu (liu.chen@zs-hospital.sh.cn) or Juan Tang (tangjuan@tongji.edu.cn) or Hui Zhang (zhanghui1@shanghaitech.edu.cn)

This pdf file includes:

Materials and Methods

Supplementary Table S1

Supplementary Videos S1-S2

Supplementary Figures S1-S17

Materials and Methods

Experimental mice

All mice were maintained on a C57BL/6 background. All mouse experiments were carried out according to the guidelines of the Institutional Animal Care and Use Committee at ShanghaiTech University.

Cell culture

RAW264.7 macrophages, HEK293T cells and mouse embryonic fibroblasts (MEF) were cultured in DMEM (Hyclone, SH30022.01) supplemented with 10% fetal bovine serum (Corning, 35-081-CV) and 1% Antibiotic-Antimycotic (Thermo, 15240062). BMDM cells were cultured in DMEM supplemented with 20% fetal bovine serum and 1% Antibiotic-Antimycotic. THP-1 cells were cultured in RPMI Medium 1640 (Invitrogen, 11875-093) supplemented with 10% fetal bovine serum and 0.05 mM β-Mercaptoethanol (Invitrogen, 21985).

AngII/PE-induced injury model

Wild-type C57BL/6 mice, aged 8-10 weeks, were randomized into different groups. The mice were anesthetized using isoflurane. AngII (1.5 μg/g/day) and PE (50 μg/g/day) were administered continuously through subcutaneous osmotic mini-pumps.

CAR plasmid construction and lentivirus production

The pHR CD19-mMegf10 CAR plasmid (Addgene, 113013) was first linearized using the restriction enzymes MluI and NotI to remove the anti-CD19 CAR sequcence, thereby producing a backbone plasmid. Subsequent homologous recombination was performed to ligate this backbone plasmid with segments of signal peptide (sp)-anti-FAP scFV-CD8 stalk/TM-mMegf10-GFP (CAR-P), sp-anti-FAP scFV-CD8 stalk/TM-GFP (CAR-C), or sp-scramble scFV-CD8 stalk/TM-mMegf10-GFP (CAR-S) using the NovoRec plus one-step PCR cloning kit (Novoprotein, NR005). The sequence information for each construct is detailed in Supplementary Table S1. These constructed CAR plasmids were subsequently packaged into

lentiviruses at OBiO Technology (Shanghai) Co., Ltd., using a four-plasmid lentiviral packaging system. This system employed the packaging plasmids pLP1 and pLP2 for the structural genes gag/pol and the regulatory gene rev, respectively, while the envelope plasmid pLP/VSVG was used to express the envelope protein VSVG.

Lentiviral infection of cultured cells

Cells were seeded the day prior to lentiviral infection to achieve approximately 50% confluency on the day of transduction. Following a culture medium change in the morning, lentiviruses with titters of 6×10^8 TU/mL (MOI: 30) were used to infect macrophages and MEF. HEK293T cells were infected with lentiviruses at titers of 3×10^8 TU/mL (MOI: 3). The viruses were added to the culture medium, thoroughly mixed, and then incubated for 12-24 hours before the medium was replaced. The efficiency of viral infection was evaluated using a fluorescence microscope 72 hours post-infection. Cells not adequately transduced were reassessed at 96-120 hours post-infection.

BMDM generation and adoptive CAR-M transfer

Mouse femurs and tibias were harvested, and surrounding muscle tissue was meticulously removed. The bones were then submerged in 75% ethanol and subsequently rinsed with PBS. Both ends of each long bone were excised to extract the bone marrow by flushing with DMEM. Following this, the marrow extract was centriguged, the supernatant discarded, and the pellet resuspended in red blood cell lysis solution. The suspension unerwent lysis for 3 minutes at room temperature and was subsequently centrifuged at 500g for 8 minutes; the supernatant was then removed. The resultant cell pellets were resuspended in 3 mL of DMEM and filtered through a 70-µm cell strainer. The cells were once again centrifuged, resuspended, and plated in culture dishes with complete media supplemented with 10 ng/mL M-CSF (R&D, 416-ML-010). Fresh medium was added on the fifth day, and mature BMDM were harvested on the seventh day. Subsequently, the BMDM cells were infected with lentiviruses at a tier of 6×10^8 TU/mL (MOI: 30) for 24 hours. 96 hours post-transduction, adoptive transfer of CAR-M cells was conducted by injecting the BMDM cells

intravenously through the tail vein at 1- and 2-week time points after the initial AngII/PE treatment. Each mouse received a single injection containing 5×10^6 cells at one time.

Quantitative real-time PCR

RNA was extracted from cells using Trizol (Magen, R4801-02) according to the manufacturer's instructions. Subsequently, the RNA was reverse-transcribed into cDNA using the PrimeScript cDNA synthesis kit (Takara, 6110A). The resulting cDNA was diluted and used for quantitative real-time PCR with SYBR Green qPCR master mix (Vazyme, Q711-03) on a StepOnePlus real-time PCR system (Applied Biosystems). Primers for detecting the mRNA levels of *anti-FAP scFV*, *Fap* and *Gapdh* are as follows. *Anti-FAP scFV*: forward 5'-CTGCAGGTCTAGTCAGAGCA-3' and reverse 5'-AATCTGTCCCAGACCCACTG-3'; *Fap*: forward 5'-ATAGCAGTGGCTCCAGTCTC-3' and reverse 5'-TCATCTGCTGTTCCGTGGAT-3'; Mouse *Gapdh*: forward 5'-TTGTCTCCTGCGACTTCAAC-3' and reverse 5'-GTCATACCAGGAAATGAGCTTG-3'; Human *Gapdh*: forward 5'-TCGGAGTCAACGGATTTGGT-3' and reverse 5'-TTCCCGTTCTCAGCCTTGAC-3'.

Latex beads phagocytosis assay

RAW264.7 macrophages were seeded into 24-well plates and allowed to reach the logarithmic growth phase. Subsequently, 1 μL fluorescent latex beads (Sigma, L3030) was added to each well. After incubating for 24 hours at 37°C, the phagocytic activity of the macrophages was assessed using a confocal microscope (Zeiss LSM 710).

Immunostaining

For tissue slides: frozen sections were air-dried at room temperature for 20-30 minutes, after which OCT compound was removed with PBS. The sections were subsequently blocked with 5% donkey serum at room temperature for 30 minutes. For cell round coverslips: Cells were initially fixed with 4% paraformaldehyde at 4°C for 15 minutes. Following fixation, the cells were washed twice with PBS and then were blocked

with 5% donkey serum at room temperature for 30 minutes. Both primary and secondary antibodies were diluted in an antibody diluent containing 2.5% donkey serum and 1% Triton. The primary antibodies were used as follows: anti-GFP (1:200, A11122, Invitrogen), anti-PDGFRa (1:500, AF1062, R&D), anti-FAP (1:100, ab28244, Abcam), anti-CD68 (1:200, MCA1957, Bio-Rad) , anti-CD3 (1:200, 14-0032-85, Invitrogen), anti-CD9 (1:100, CY5337, ABways), anti-MPO (1:100, AF3667-SP, R&D), anti-ARG1 (1:100, 93668T, Cell Signaling Technology), anti-TNNI3 (1:100, ab56357, Abcam), anti-CDH5 (1:100, AF1002, R&D). Tissue sections and cultured cells were incubated with primary antibodies overnight at 4°C. Post primary antibody incubation, they were washed with PBS and incubated with Alexa Fluor conjugated secondary antibodies (Invitrogen) at room temperature for 1 hour. Subsequent to incubation, slides and coverslips were washed with PBS and stained with DAPI for nucleus idenfitication. Images were acquired using confocal microscopes (Nikon A1R, Zeiss LSM 710, Zeiss LSM 800 and Zeiss LSM 980).

THP-1-derived macrophages

THP-1 cells in the logarithmic growth phase were centrifuged and resuspended in RPMI-1640 medium. The cell density was adjusted to 5×10^5 cells/mL, with a final PMA (Beyotime, S1819) concentration of 100 ng/mL. The cells were then seeded into six-well plates at a volume of 2 mL per well. After 48 hours, successful differentiation into human macrophages was confirmed by microscopic observation.

Isolation of mouse embryonic fibroblasts

Mouse embryos between embryonic days 12.5-14.5 were dissected from pregnant mice, and the heads, tails, internal organs, and limbs were removed, leaving only the trunks. Each dissected was washed in PBS to remove blood and then finely minced in a dish. The minced tissues were transferred to a 50 mL centrifuge tube, and 10 mL 0.25% trypsin-EDTA (Thermo, 25200072) was added. The tissue fragments were then gently pipeted up and down 100 times. Subsequently, 3 mL fetal bovine serum was added to terminate the digestion process. The mixture were centrifuged at 1000 rpm for 5 minutes. After carefully aspirating the

supernatant, the cell pellet was resuspended in DMEM supplemented with 10% fetal bovine serum and seeded into a 10 cm dish. Primary MEF were used between passage 0 and 3 for subsequent experiments.

Quantitative analysis of internalized vesicles and live-cell imaging

Macrophages and target cells were co-cultured in a 24-well plate using complete culture medium at an initial ratio of 1:2. After an 8-hour incubation, the cells were observed and counted using a confocal microscope (Nikon TI2-E+CSU W1 and Olympus SpinSR10). Live-cell imaging was performed using the confocal microscope (Nikon TI2-E+CSU W1), with images acquired every 3 minutes over a total duration of 16 hours.

Flow cytometry

Peripheral blood samples were collected from the orbital vein of mice into tubes containing ACD anticoagulant (MeilunBio, MA0199). For heart samples, cardiac tissues were harvested, finely minced, and digested with leberase TH (Roche, 5401151001) to facilitate dissociation. Both peripheral blood and heart samples were treated with red blood cell lysis buffer (Beyotime, C3702) prior to flow cytometry analysis. The isolated $BMDM^{CAR-P}$ cells were stained with APC-anti-Ly6c (1:100, 560595, BD Biosciences) for 1 hour at 4°C. The various cell populations were subsequently gated and analyzed using flow cytometers (BD Fortessa and Beckman CytoFLEX S).

Picro-Sirius red staining

Picro-Sirius red staining was conducted using a kit (Abcam, 150681) according to the manufacturer's instruction. Briefly, frozen sections were air dried at room temperature for 20 minutes and then rinsed with distilled water to remove OCT. The tissue sections were immersed in picro-sirius red solution and incubated at room temperature for 1 hour. Following the incubation, the slides were washed twice with acetic acid solution, then twice with anhydrous ethanol, and finally twice with xylene before cover slips were mounted. Images were obtained using a stereomicroscope (Olympus MVX10).

H&E staining

Sections were air-dried for 30 minutes and washed under running water for 5 minutes to remove OCT. The sections were then stained wiht hematoxylin solution for 10 minutes, followed by a rinse under running water to remove excess dye. Then, the sections were immersed in 0.1% hydrochloric ethanol for 1.5 minutes and rinsed again under running water. Next, sections were placed in PBS for bluing, rinsed once more with running water, and finally, mounted with neutral resin. Images were obtained using an a microscope (Olympus CX23).

Serum cytokine assay

Blood was collected from the orbital vein of mice at 4 and 9 weeks following the initial AngII/PE treatment. Cytokine levels were measured using a mouse cytokine 25plex chip (EMD Millipore, mcytomag-70k-PMX) by multiplex technology (Luminex, MAGPIX).

ELISA for IL-6 quantification

Peripheral blood was collected from mice 4 weeks after the initial AngII/PE treatment and allowed to clot for 30 minutes. Then, the samples were centrifuged at 1000g for 10 minutes, and the supernatant serum was collected for subsequent analysis. The level of IL-6 were measured using a IL-6 ELISA kit (Yeasen, 98027ES48) according to the manufacturer's instruction. Briefly, the ELISA plate was conditioned by soaking for 30 seconds and then thoroughly dried. Standards and serum samples were added to the designated wells, and the plate was incubated for 2 hours at room temperature. Following the incubation, the liquid were discarded, and the plate was dried. Detection antibodies, diluted to the appropriate working concentration, were then added, and the plate was subjected to another 2-hour incubation at room temperature. The liquid was again discarded and the plate dried before the addition of the enzyme conjugate, prepared at its working concentration, for a 20-minute incubation at room temperature. After repeating the discard and dry steps, the substrate solution was applied, and the plate was incubated in darkness at room

temperature for 15 minutes. The reaction was halted by adding the stop solution, and the absorbance was measured at 450 nm/630 nm within 10 minutes.

Statistics analysis

Data were representative of at least three independent experiments and are presented as mean values ± SEM. Statistical significance was determined using Student's t-test (for comparison between two groups) and one-way or two-way ANOVA followed by Tukey's multiple comparison test (for comparisons among three or more groups). Significance was accepted when $p < 0.05$. The statistical analyses were conducted by investigators blinded to animal group assignments.

Supplementary Videos S1-S2

S1: The nibbling process of $RAW^{CAR\text{-}P}$ macrophages towards $293T^{FAP\text{-}mCherry}$ cells.

S2: $RAW^{CAR\text{-}P}$ macrophages engulfed entire $293T^{FAP\text{-}mCherry}$ cells.

Supplementary Table S1

Detailed information for each construct of the CAR molecule

Designation	Information
Signal peptide	Amino acid 1-21 CD8 (Uniprot Q96QR6_HUMAN)
Anti-FAP scFV V-L chain	Amino acid sequence:<br>DVLMTQTPLWLPVSLGDQASISCRSSQSIVHSNGNTYLEWYLQKPGQSPK<br>LLIYKVSNRFSGVPDRFSGSGSGTDFTVKISRVEAEDLGVYYCFGGSHVPY<br>TFGGGTKLEIK
Anti-FAP scFV V-H chain	Amino acid sequence:<br>QVQLKESGGLVQPGGSLKLSCAASGFTFSSYGMSWVRQTADKRLELVAT<br>TNNNGGVTYYPDSVKGRFTISRDNAKNTLYLQMSSLQSEDTAMYYCARY<br>GYYAMDYWGQGISVTVSS
CD8 stalk/TM	Amino acid 138-206 CD8 (Uniprot Q96QR6_HUMAN)
Megf10 cytosolic domain	Amino acid 879-1147 Mouse Megf10 (Uniprot Q6DIB5(MEG10_MOUSE))
Scrambled scFV V-L chain	Amino acid sequence:<br>GNVKQTIKRHSGVGSKTVYASLGVYQSTQYKTHVPCLMSGDDDGDKLEL<br>IFLARGKDYFQGRPESPFGSEWYLYVLRNGPFVPSFWLGGIVTIEQPLSNST<br>SLSISGTVSSC
Scrambled scFV V-H chain	Amino acid sequence:<br>TQKGVASDQKATMLKQYVNTSSILLVTTGYATSYVTSEYVWCGANDGFK<br>GTQYGSSTSAGSSADASYLRKMPFGFYWSQNGLADLLEYRSRPNVSYMY<br>RNGQELLDGVSQCMIRGV

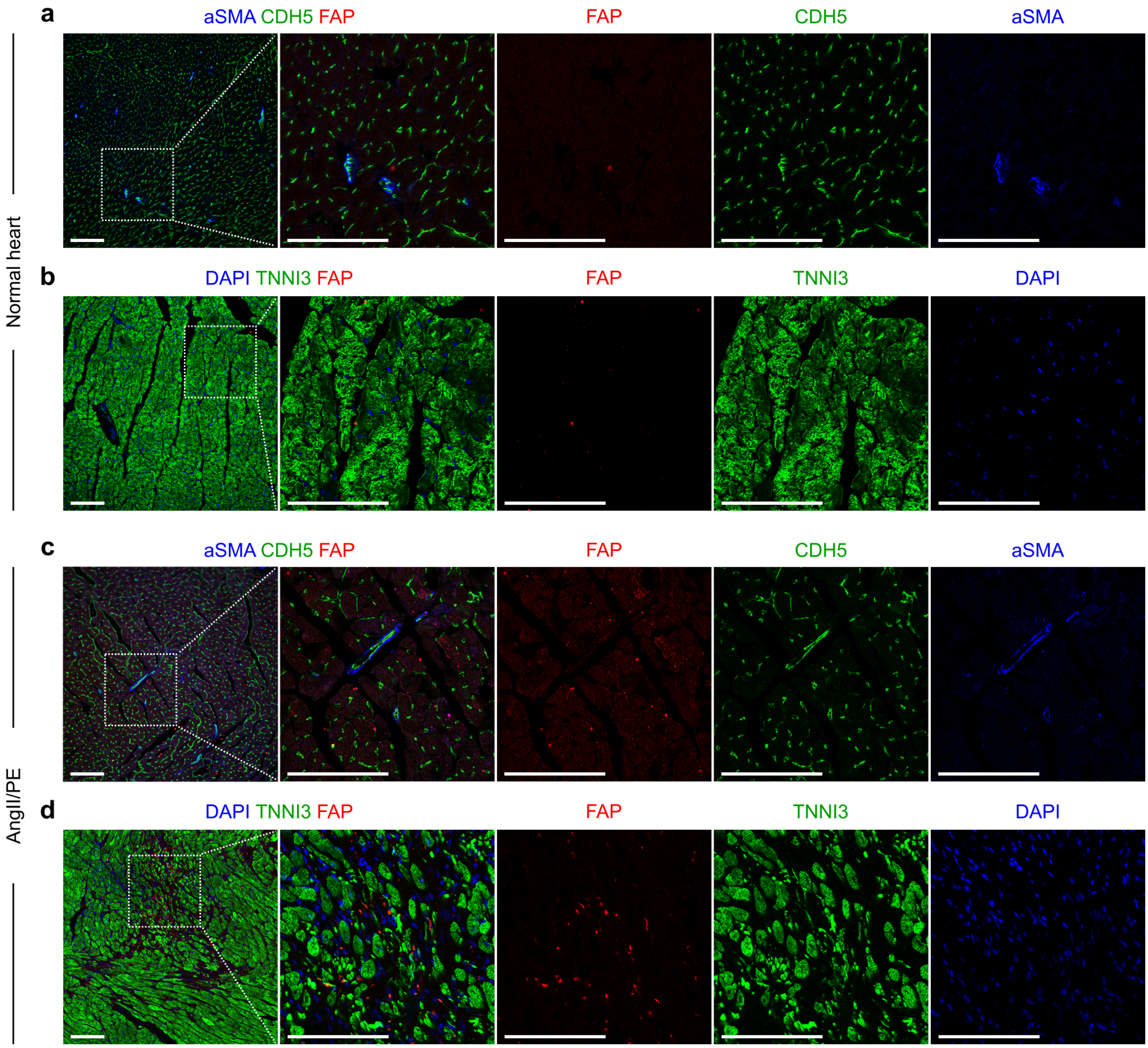

Supplementary Fig. S1. FAP is not expressed in coronary vascular endothelial cells, smooth muscle cells, or cardiomyocytes in mouse hearts, with or without AngII/PE treatment. **a** and **b** Few FAP⁺ cells were identified in adult normal hearts. FAP is not detected in coronary vascular endothelial cells (**a**), smooth muscle cells (**a**), or cardiomyocytes (**b**) in adult normal hearts. **c** and **d** FAP is not detected in coronary vascular endothelial cells (**c**), smooth muscle cells (**c**), or cardiomyocytes (**d**) in AngII/PE-treated hearts. Scale bars, 100 μm. Each picture is representative of four samples.

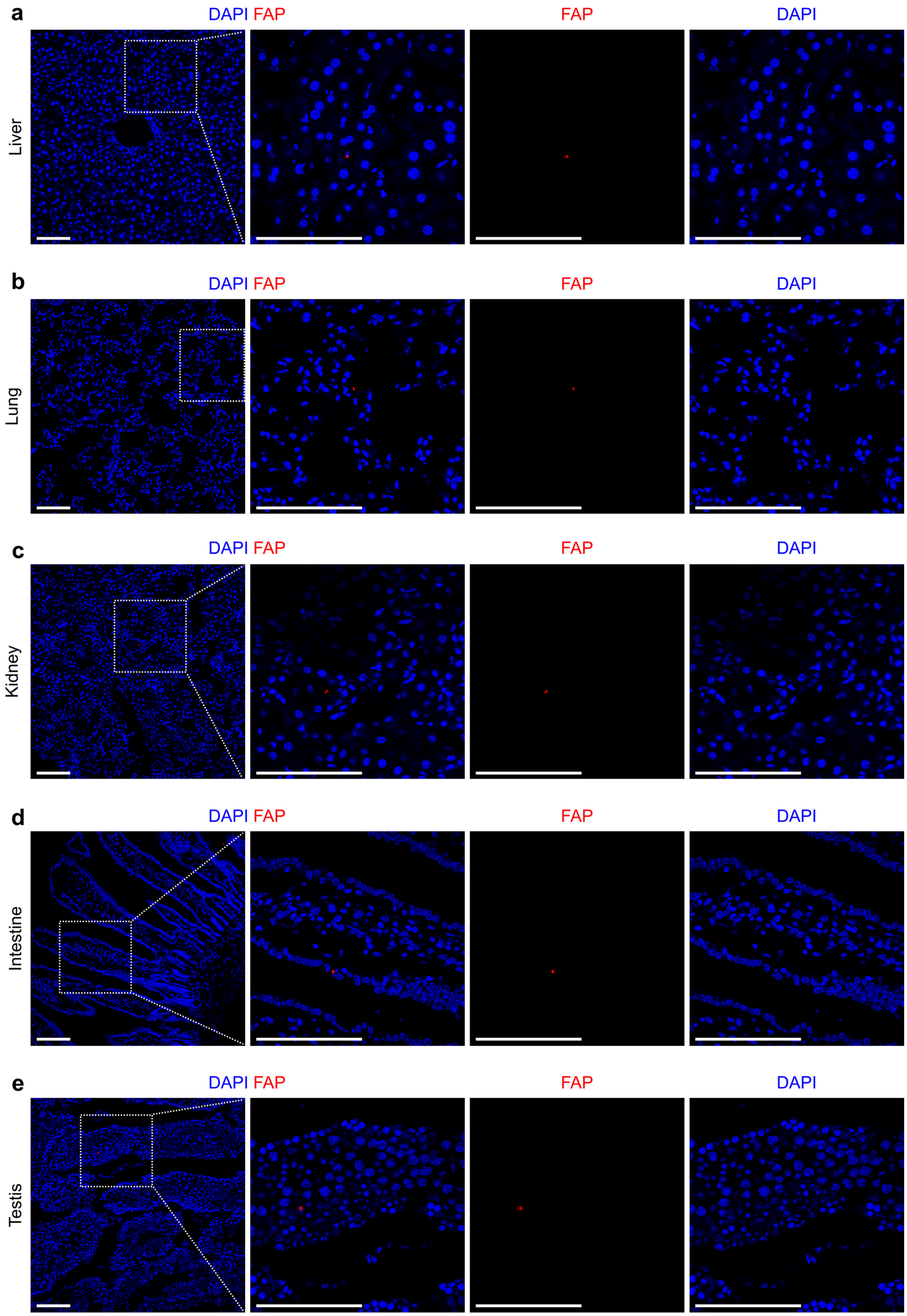

Supplementary Fig. S2. Few FAP⁺ cells are identified in the liver, lung, kidney, intestine or testis of AngII/PE-treated mice. a–e Immunostaining for FAP on cryosections from various organs taken four weeks after the initial AngII/PE treatment. Scale bars, 100 μm. Each picture is representative of four samples.

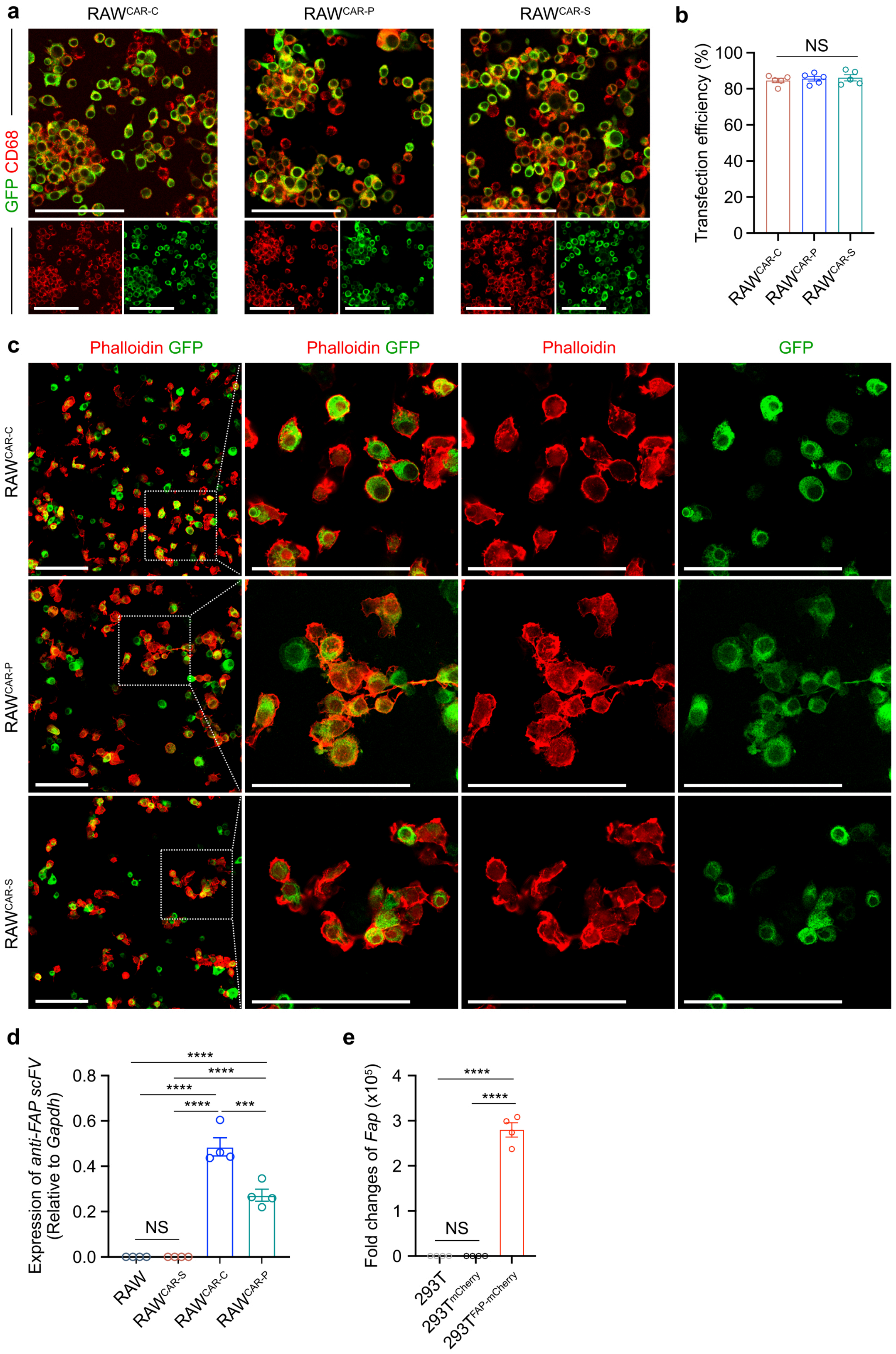

Supplementary Fig. S3. Characterisation of CAR expression in RAW264.7 cells and FAP expression in HEK293T cells. a GFP expression in CAR-expressing RAW264.7 cells. Scale bars, 100 μm. **b** Transfection efficiency of RAW264.7 cells. Each group contains five independent experiments. Statistical significance was analysed using one-way ANOVA. NS, non-significant. **c** GFP expression along the inner side the plasma membrane in RAW264.7 cells. Phalloidin staining is used to visualise cell membrane. Scale bars, 100 μm. **d** The relative mRNA levels of *anti-FAP scFV* in RAW264.7 cells. n = 4 per group. Statistical significance was analysed using one-way ANOVA. NS, non-significant; *** $p < 0.001$; **** $p < 0.0001$. **e** The relative mRNA levels of *Fap* in HEK293T cells. n = 4 per group. Statistical significance was analysed using one-way ANOVA. NS, non-significant; **** $p < 0.0001$.

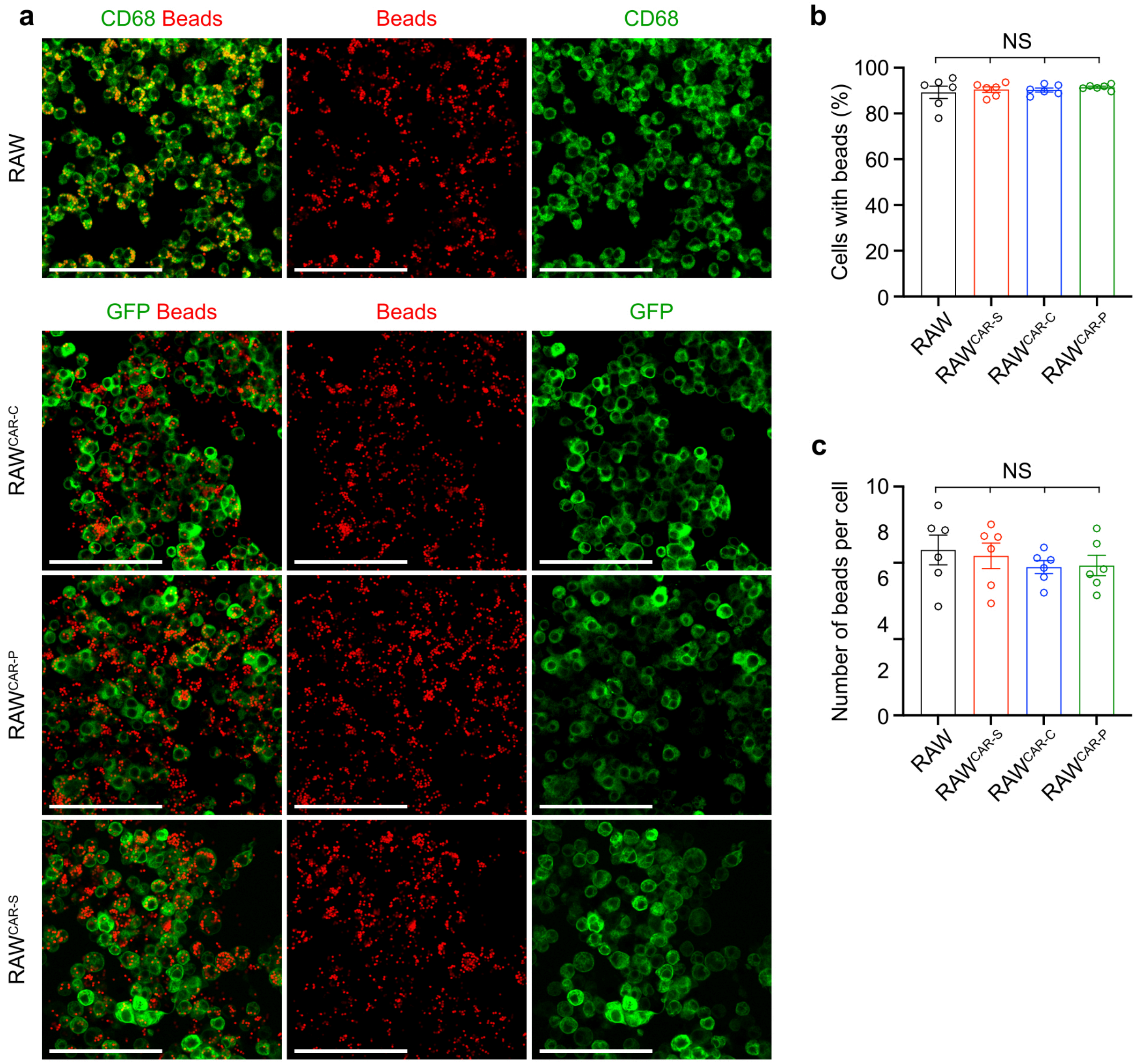

Supplementary Fig. S4. CAR expression does not affect general phagocytic function of RAW264.7 cells. a Co-culture of RAW264.7 cells with carboxylate-modified red fluorescent latex beads. CD68 staining is used to identify the wild-type RAW264.7 cells, and GFP staining denotes the CAR-expressing RAW264.7 cells. Scale bars, 100 µm. **b** Percentage of $CD68^+$ or GFP^+ RAW264.7 cells with fully internalized beads. Differences were analysed using one-way ANOVA. Each group comprises data from six independent experiments. NS, non-significant. **c** Number of phagocytosed beads per RAW264.7 cell. Each group contains data from six independent experiments. Differences were analysed using one-way ANOVA. NS, non-significant.

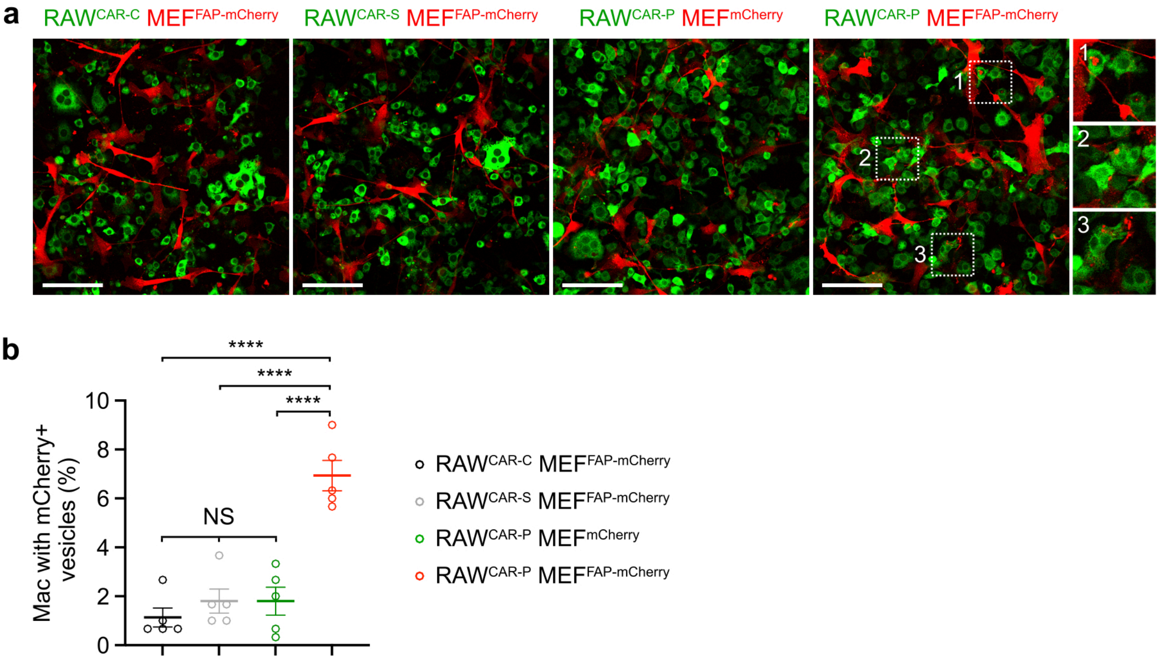

Supplementary Fig. S5. RAW^{CAR-P} engulfment of FAP^{+} fibroblasts *in vitro*. a Fully internalised mCherry^{+} vesicles were identified in RAW^{CAR-P} cells co-cultured with MEF$^{FAP-mCherry}$ cells. Scale bars, 100 μm. **b** Percentage of RAW264.7 cells with internalised mCherry^{+} vesicles. Each group contains five independent experiments. Statistical significance was analysed using one-way ANOVA. NS, non-significant; **** p < 0.0001.

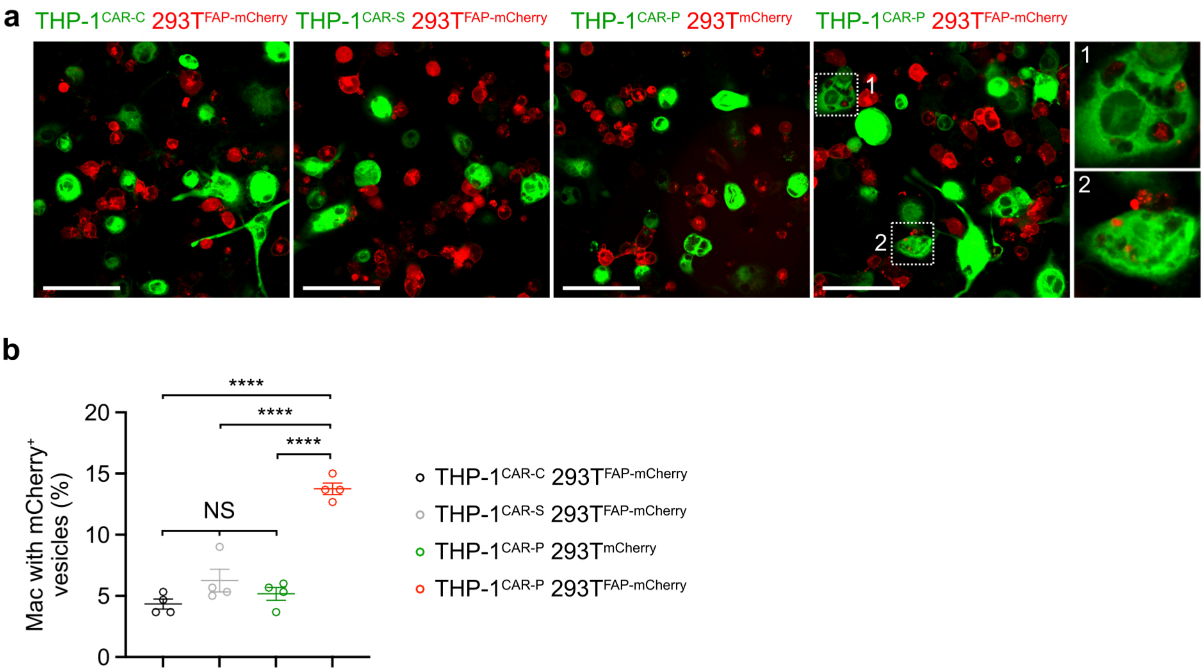

Supplementary Fig. S6. Engulfment of FAP$^+$ target cells by THP-1$^{CAR\text{-}P}$ *in vitro*. a Fully internalized mCherry$^+$ vesicles were observed in THP-1$^{CAR\text{-}P}$ cells co-cultured with 293T$^{FAP\text{-}mCherry}$ cells. Scale bars, 100 µm. **b** Percentage of THP-1 cells containing internalized mCherry$^+$ vesicles. Data from four independent experiments are presented. Differences were analysed using one-way ANOVA. NS, non-significant; **** $p < 0.0001$.

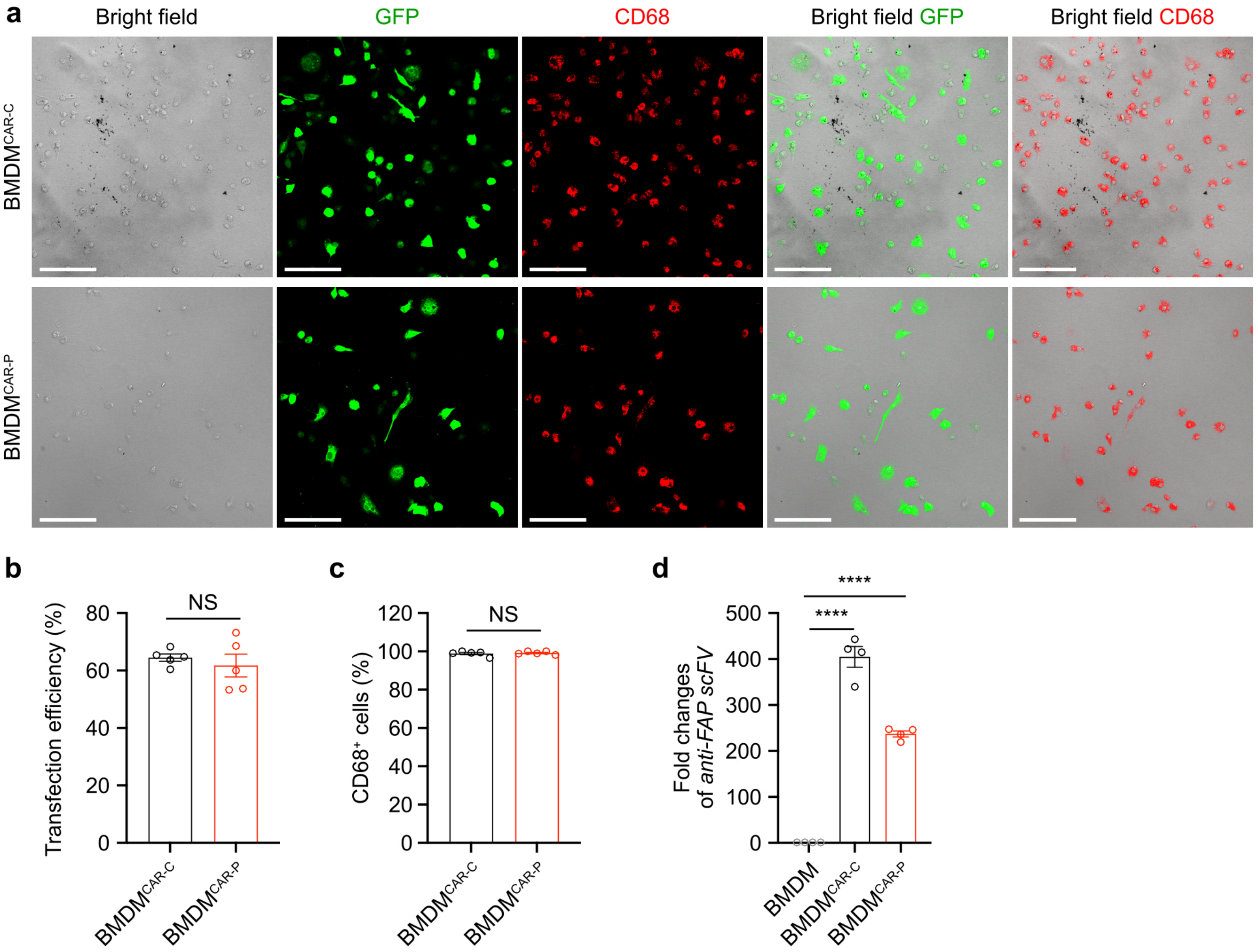

Supplementary Fig. S7. Characterisation of BMDM^{CAR}. **a** Expression of GFP and CD68 in BMDM^{CAR-C} and BMDM^{CAR-P} cells. Scale bars, 100 μm. **b** Percentage of GFP^{+} cells in BMDM^{CAR-C} and BMDM^{CAR-P} groups. n = 5 per group. Difference analysed using Student's t-test. NS, non-significant. **c** Percentage of CD68^{+} cells in BMDM^{CAR-C} and BMDM^{CAR-P} groups. n = 5 per group. Difference analysed using Student's t-test. NS, non-significant. **d** Fold changes in *anti-FAP scFV* expression in BMDM cells. Difference analysed using one-way ANOVA. **** p < 0.0001. n = 4 per group.

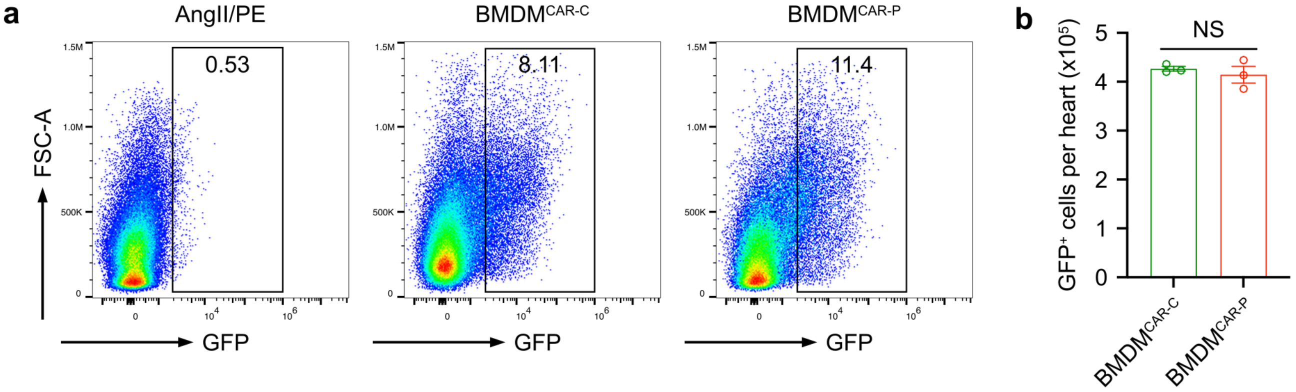

Supplementary Fig. S8. Detection of GFP⁺ cells in the heart three days after the final BMDM^{CAR} injection. a FACS analysis reveals the presence of GFP⁺ cells in the hearts three days post the final BMDM^{CAR} injection. **b** Quantification of GFP⁺ cells per heart at the same time point. n = 3 per group. Student's t test was used to determine the significance. NS, non-significant.

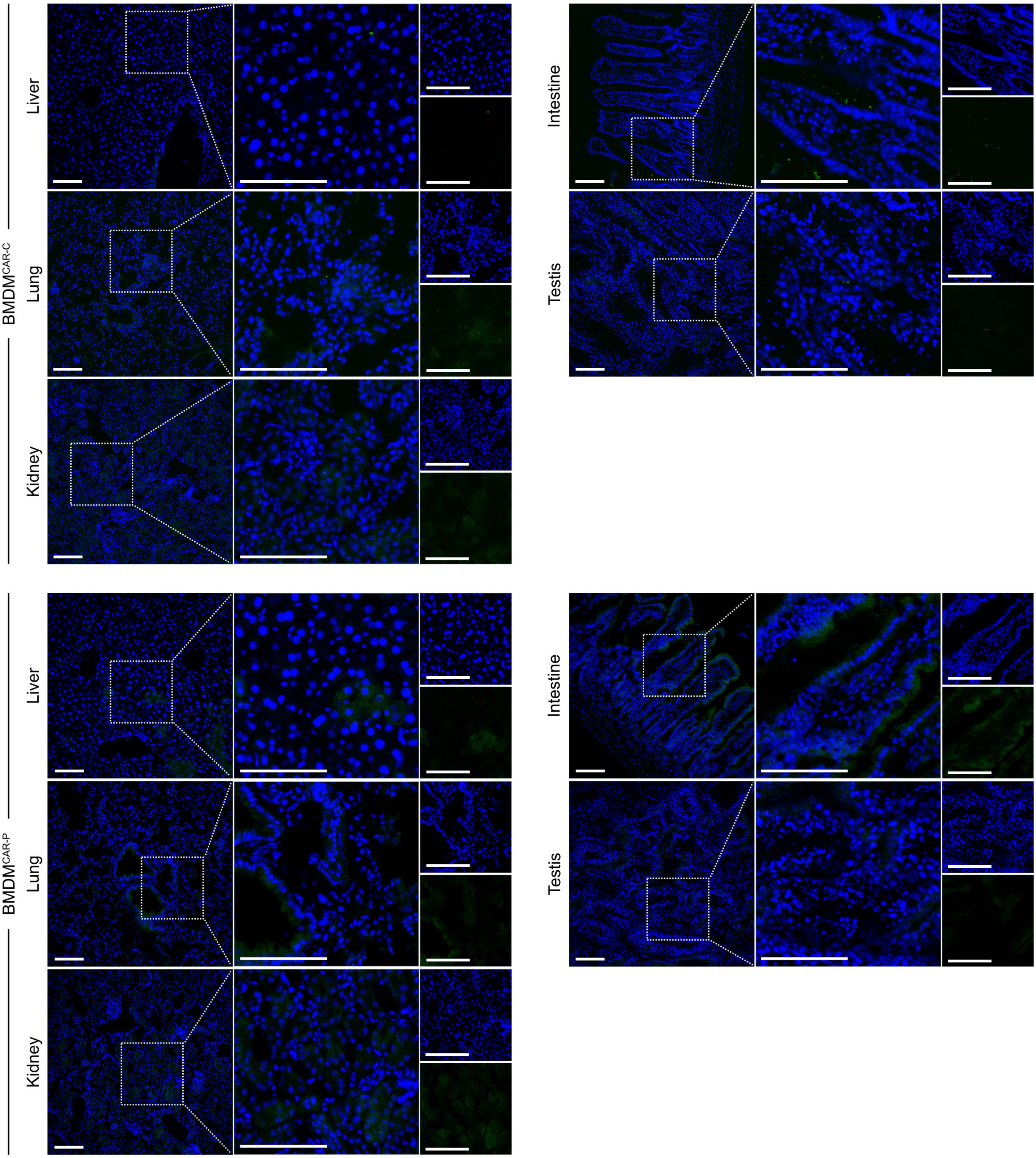

Supplementary Fig. S9. Few BMDM^{CAR-C} and BMDM^{CAR-P} cells were identified in non-cardiac organs four weeks after the initial AngII/PE treatment. GFP staining was performed on sections of the liver, lung, kidney, intestine, and testis. Five mice were examined per group. Scale bars, 100 μm.

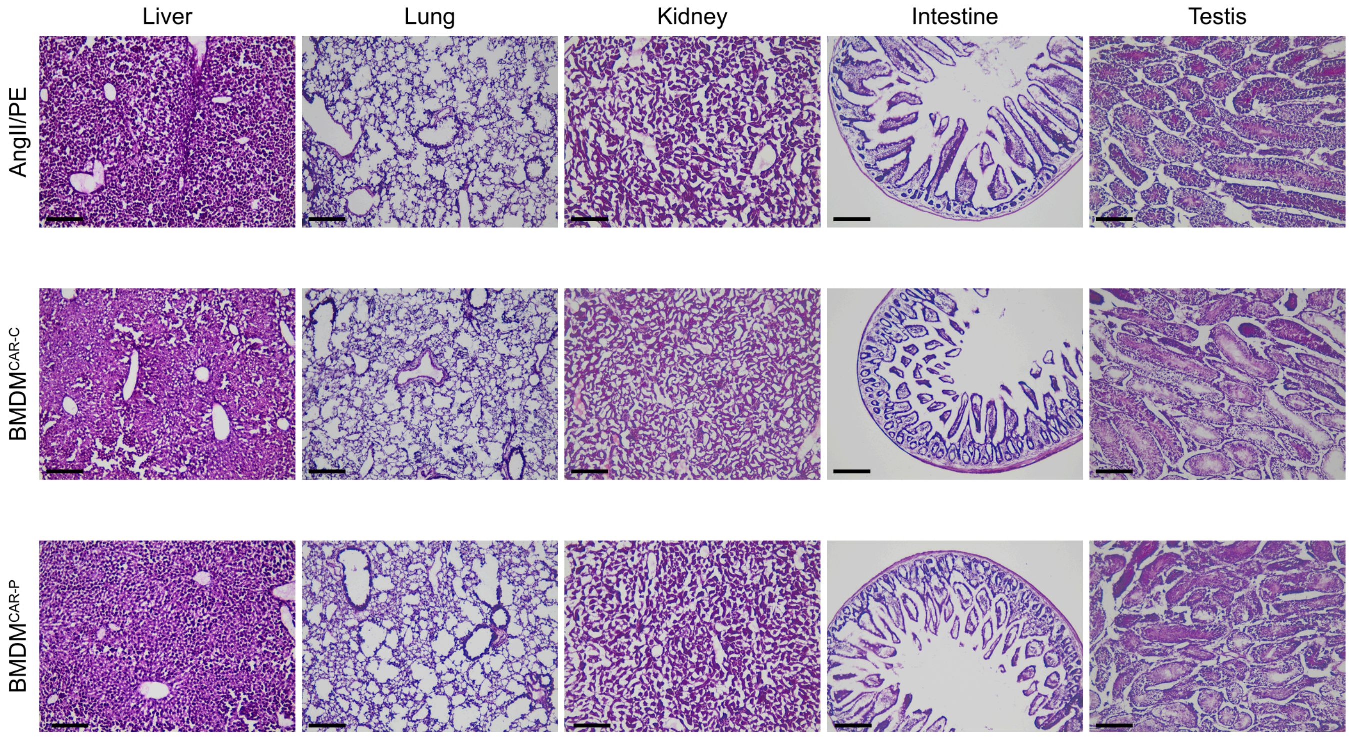

Supplementary Fig. S10. Assessment of toxicity following BMDM^{CAR} treatment. H&E staining of tissue sections from various organs of mice four weeks after the initial AngII/PE treatment. Each image is representative of five samples. Scale bars, 200 μm.

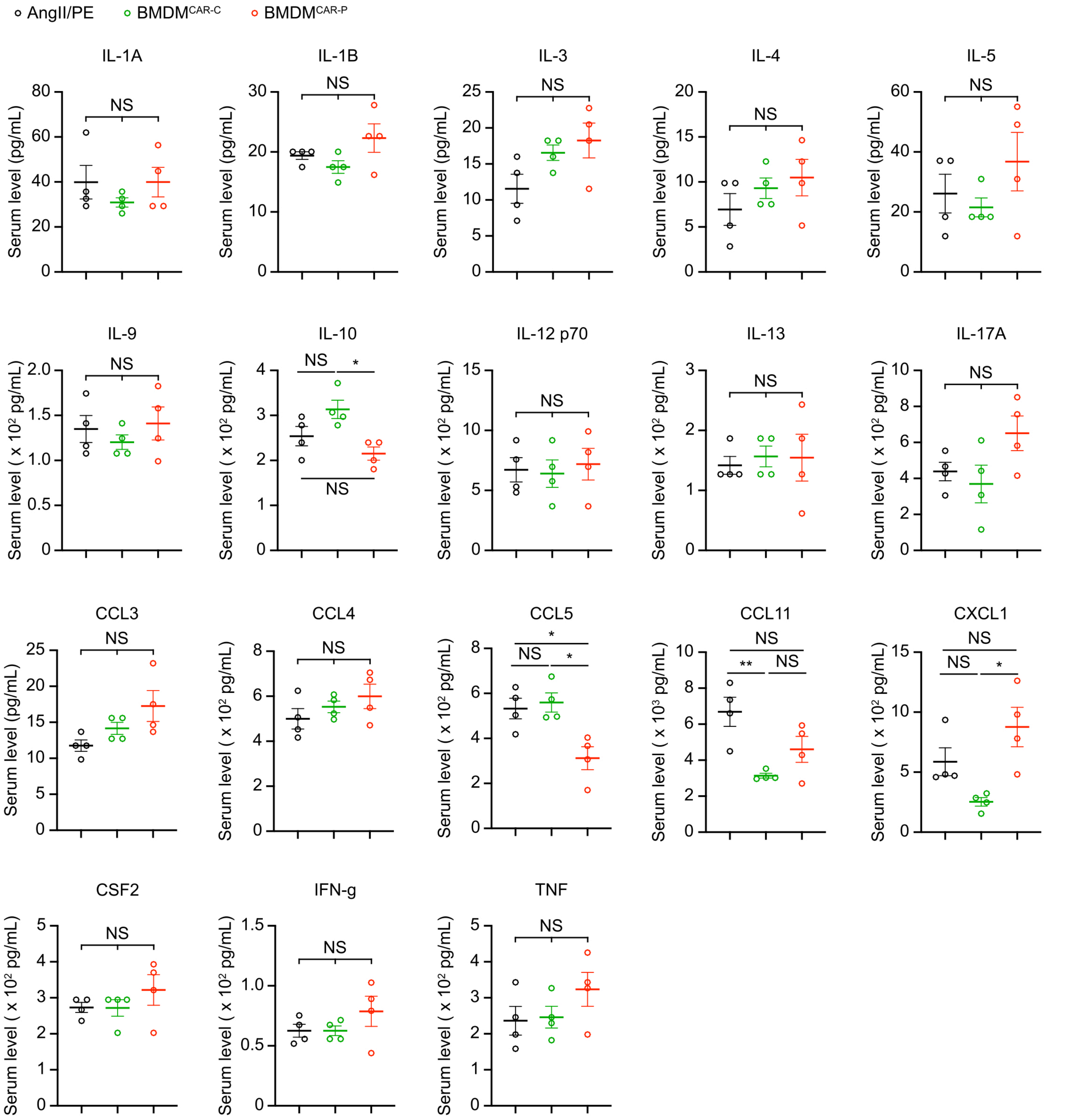

Supplementary Fig. S11. Cytokine levels in the serum of mice four weeks following the initial AngII/PE treatment. n = 4 mice per group. Differences were analysed using one-way ANOVA. NS, non-significant; * $p < 0.05$; ** $p < 0.01$.

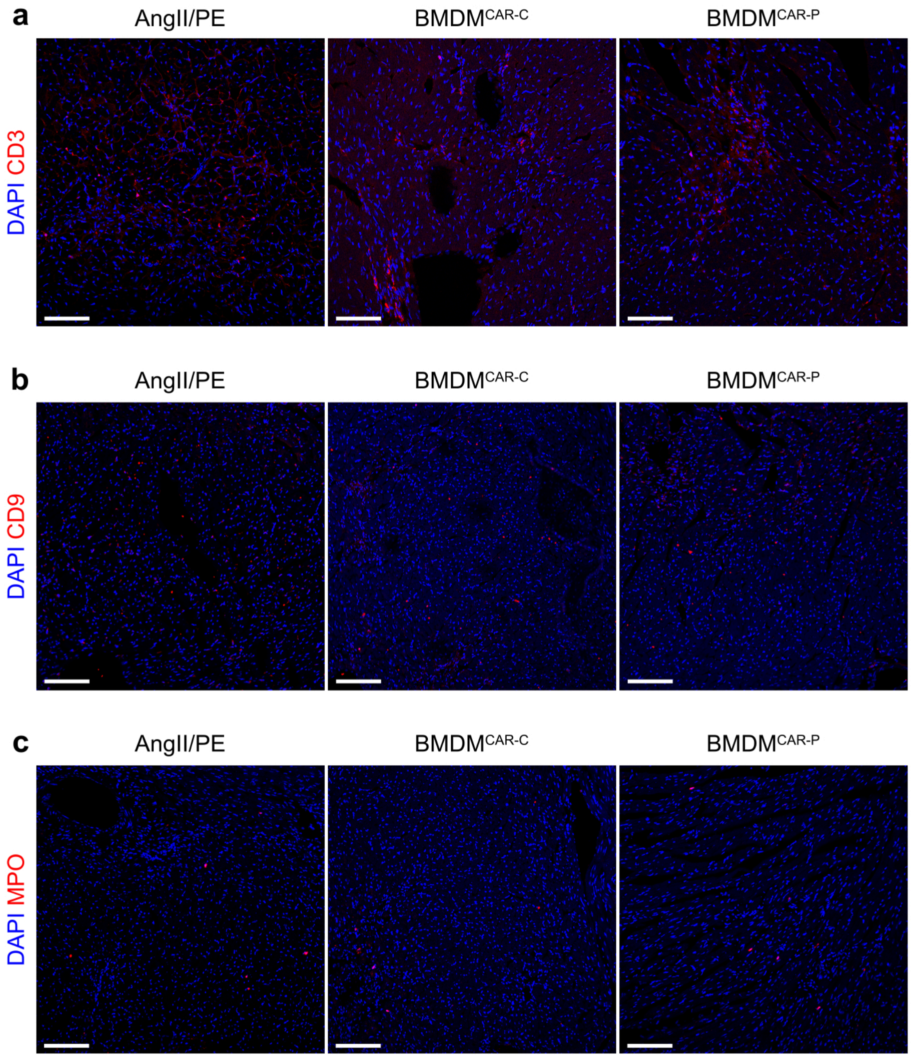

Supplementary Fig. S12. CAR-M treatment does not promote additional immune cell infiltration into the heart four weeks following the initial AngII/PE treatment. a–c Immunostaining for CD3, CD9, and MPO on heart sections from mice four weeks after AngII/PE treatment. Scale bars, 100 μm. Each picture is representative of 5 samples.

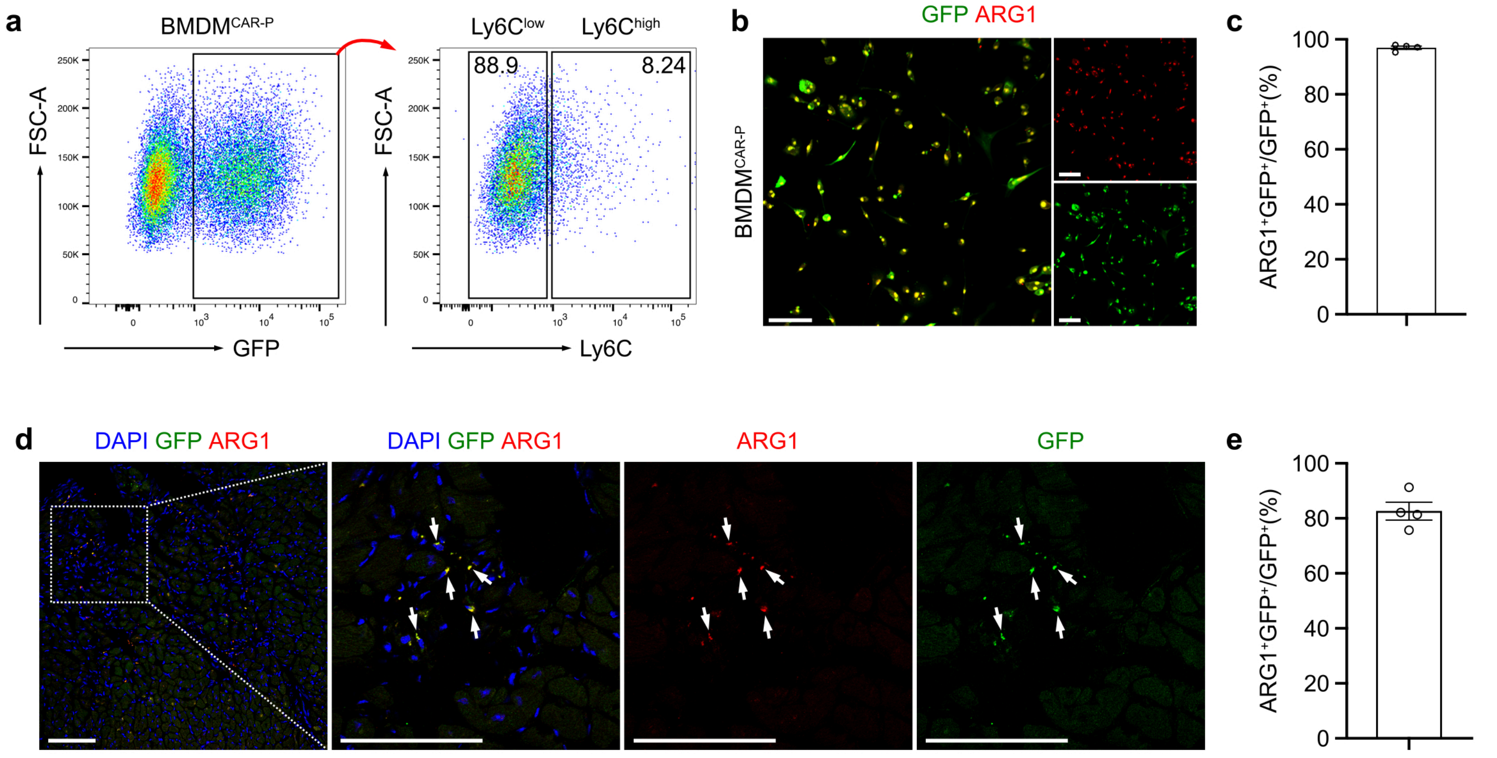

Supplementary Fig. S13. **Characterisation of BMDM^{CAR-P} are M2-like macrophages.** **a** FACS analysis of cultured BMDM^{CAR-P}. **b** Immunostaining for ARG1 and GFP on cultured BMDM^{CAR-P}. Scale bars, 100 μm. **c** Percentage of ARG1$^+$ BMDM^{CAR-P} cells in culture. n = 4. **d** Immunostaining for GFP and ARG1 on heart sections from mice four weeks after the initial AngII/PE treatment. Arrows indicate ARG1$^+$GFP$^+$ cells. Scale bars, 100 μm. **e** Percentage of ARG1$^+$ CAR-M in the hearts. n = 4 individual mouse samples.

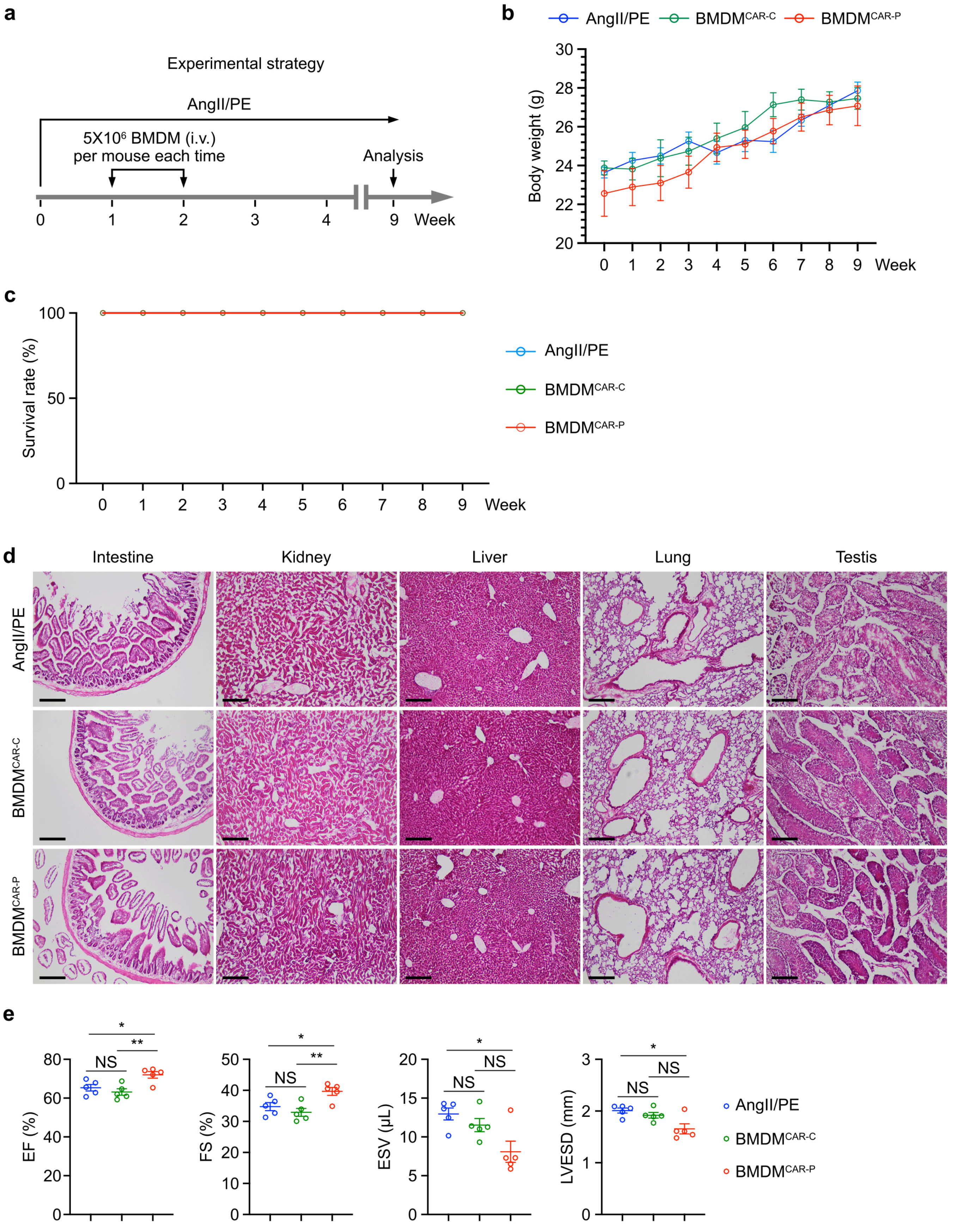

Supplementary Fig. S14. Evaluation of BMDM^{CAR} treatment toxicity over an extended period. a Experimental strategy. **b** Body weights of mice were recorded following AngII/PE and BMDM^{CAR} treatment. Each group contained five mice. Two-way ANOVA analysis revealed no significant difference among these three groups. **c** Survival rate of mice following AngII/PE and BMDM^{CAR} treatment. Each group contained ten mice. **d** H&E staining of various organ sections from mice. Scale bars, 200 μm. **e** Comparison of cardiac function parameters between experimental and control groups. Each group contained five mice. NS, non-significant; * $p < 0.05$; ** $p < 0.01$. One-way ANOVA analysis was used to determine the significance of differences.

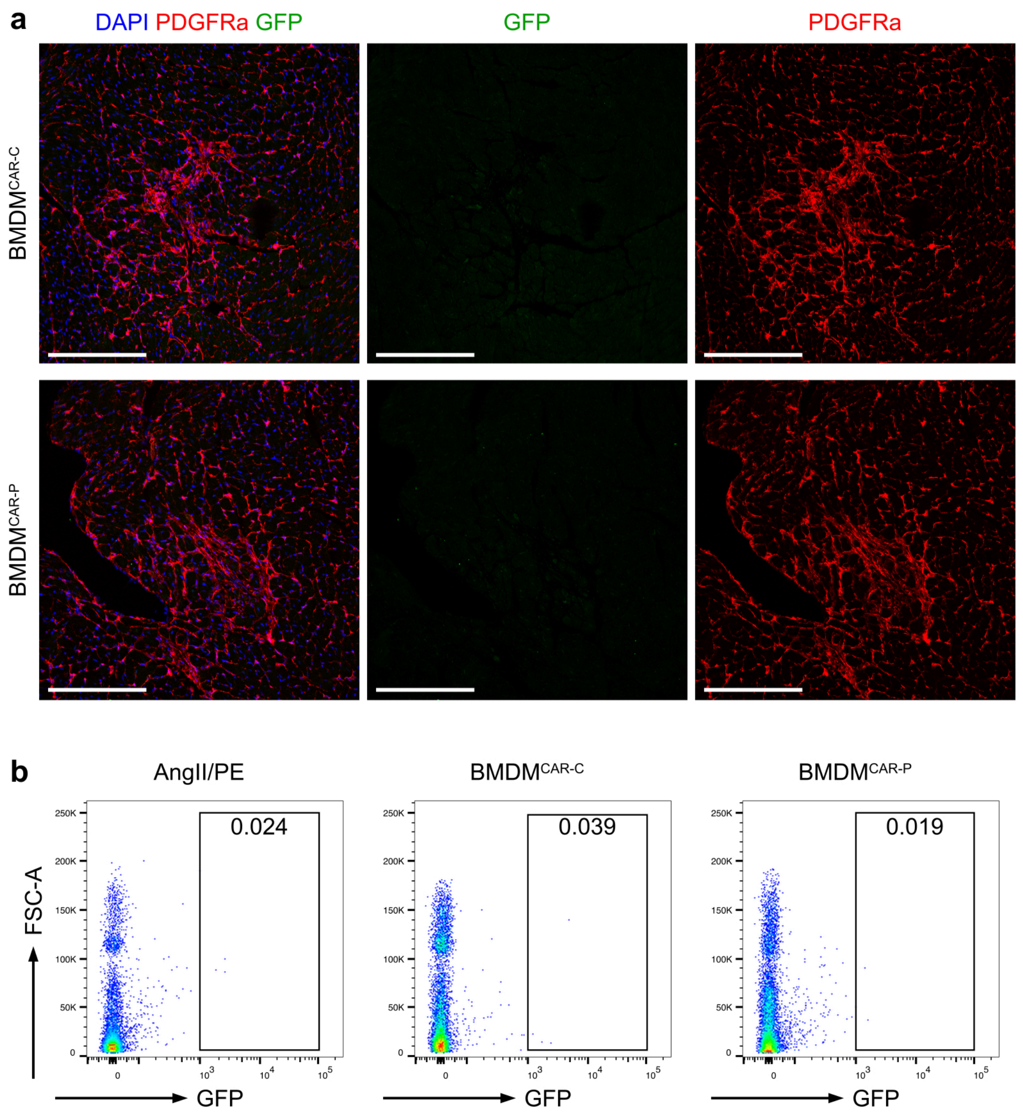

Supplementary Fig. S15. Few GFP⁺ CAR-M cells were detected in the injured hearts nine weeks after the initial AngII/PE treatment. a GFP and PDGFRa staining of heart sections nine weeks after the initial AngII/PE treatment. Scale bars, 200 μm. Five mice were examined per group. **b** FACS analysis reveals few GFP⁺ cells in mouse blood nine weeks after the initial AngII/PE treatment. n = 3 per group.

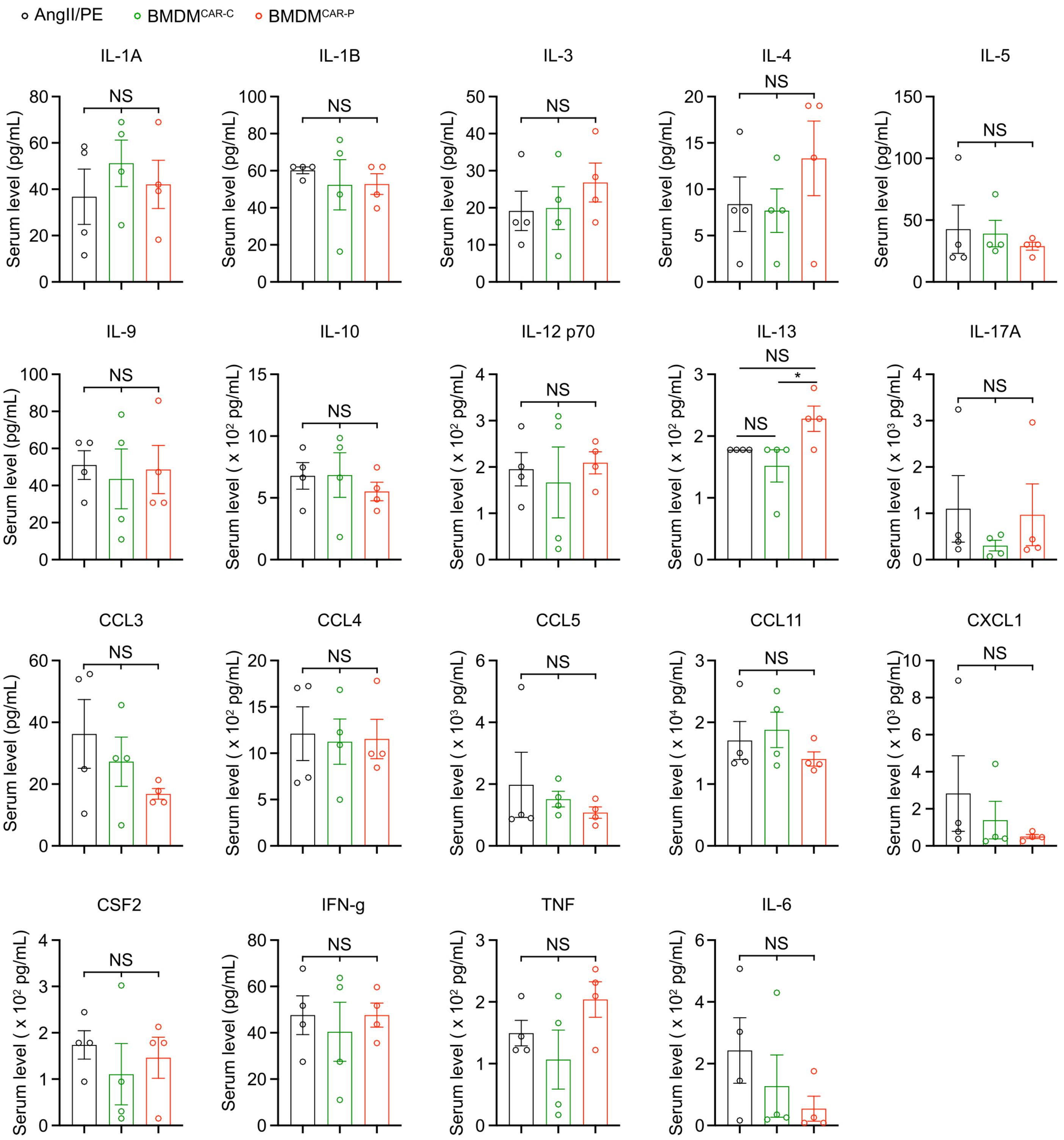

Supplementary Fig. S16. Cytokine levels in the serum of mice nine weeks following the initial treatment of AngII/PE. Each group contains four mice. Differences were analysed using one-way ANOVA. NS, non-significant; * $p < 0.05$.

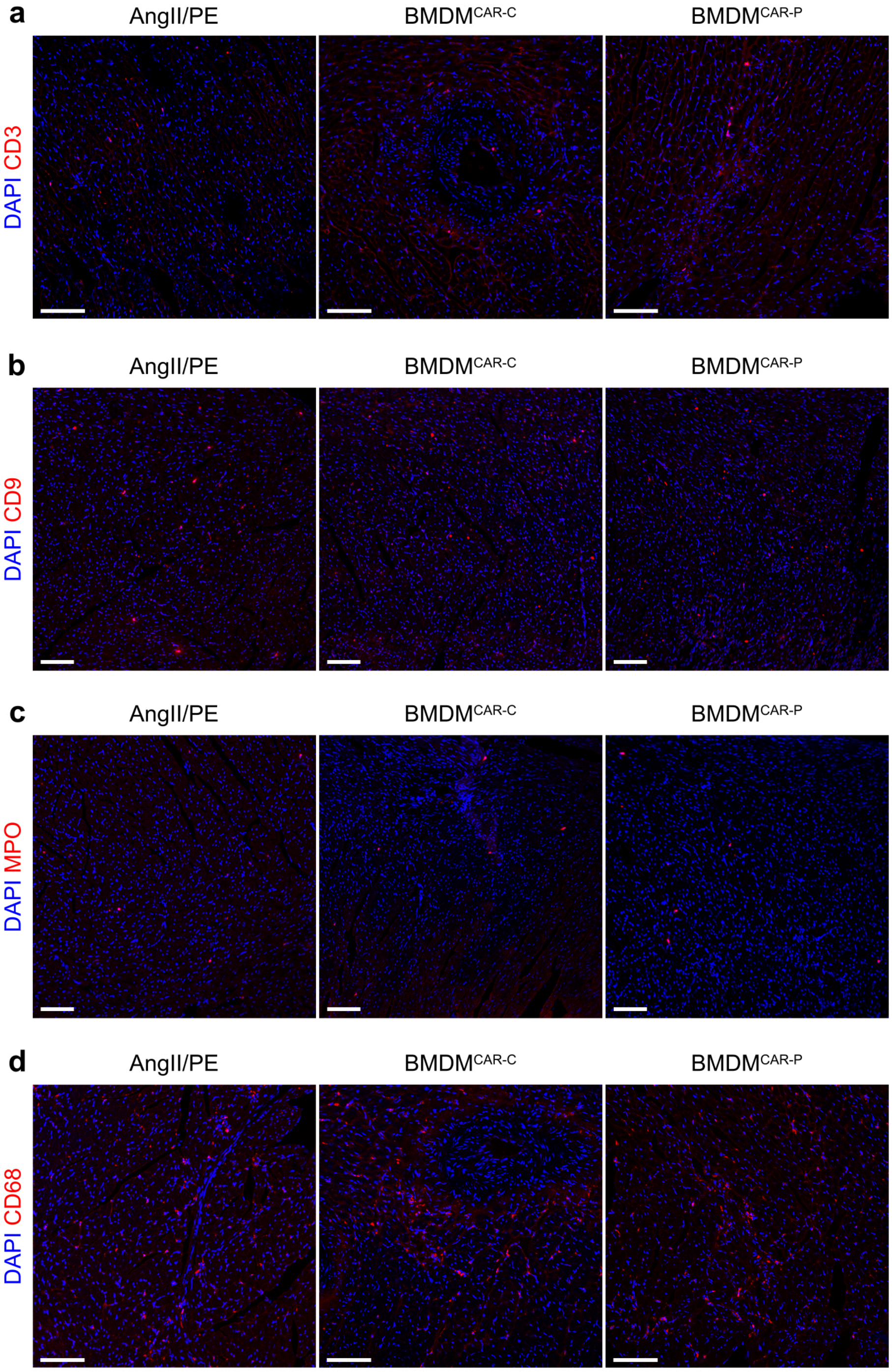

Supplementary Fig. S17. CAR-M treatment does not enhance immune cell infiltration into the heart at nine weeks after the initial AngII/PE treatment. a–d Immunostaining for CD3, CD9, MPO and CD68 on heart sections from mice nine weeks after AngII/PE treatment. Scale bars, 100 µm. Each picture is representative of five samples.
